# Effects of Cornus and Its Mixture with Oregano and Thyme Essential Oils on Dairy Sheep Performance and Milk, Yoghurt and Cheese Quality under Heat Stress

**DOI:** 10.3390/ani11041063

**Published:** 2021-04-08

**Authors:** Konstantinos Kalaitsidis, Erasmia Sidiropoulou, Olga Tsiftsoglou, Ioannis Mourtzinos, Thomas Moschakis, Zoitsa Basdagianni, Stylianos Vasilopoulos, Styliani Chatzigavriel, Diamanto Lazari, Ilias Giannenas

**Affiliations:** 1 Laboratory of Nutrition, School of Veterinary Medicine, Aristotle University of Thessaloniki, 54124 Thessaloniki, Greece; kalaitsidisf92@gmail.com (K.K.); esidiropoulou@vet.auth.gr (E.S.); svasilopoulos@vet.auth.gr (S.V.); 2 Laboratory of Pharmacognosy, School of Pharmacy, Aristotle University of Thessaloniki, 54124 Thessaloniki, Greece; olga@tsiftsoglou.gr (O.T.); dlazari@pharm.auth.gr (D.L.); 3 Department of Food Science and Technology, School of Agriculture, Aristotle University of Thessaloniki, 54124 Thessaloniki, Greece; mourtzinos@agro.auth.gr (I.M.); tmoschak@agro.auth.gr (T.M.); 4 Department of Animal Production, School of Agriculture, Aristotle University of Thessaloniki, 54124 Thessaloniki, Greece; basdagianni@agro.auth.gr; 5 ORIZON Diatrofiki, 62100 Serres, Greece; stella@orizon-ate.gr

**Keywords:** cornus, oregano, thyme, sheep, milk, heat stress

## Abstract

**Simple Summary:**

Various plant extracts have been used as feed additives to benefit ruminant performance. In this study we investigated the effects of dietary cornus plant extract supplementation, with or without the addition of oregano and thyme essential oil, in dairy ewes. Ewes participating in this experiment were reared under thermal stress and their performance, as well as the composition of their final products (milk, feta cheese and yoghurt), were assessed. The outcome of this experiment showed that cornus plant extract, alone or in combination with oregano and thyme, favored the production of ewe’s milk, along with the composition of milk and milk products.

**Abstract:**

The effect of a diet supplemented with a novel cornus extract, enriched with essential oils of oregano and thyme, on the performance of Chios cross-bred dairy sheep was investigated during the summer period. The plant extracts were prepared using a “green” method based on aqueous extraction. A total of 45 lactating ewes were allocated into three equal groups in a randomized block design. The three groups were fed the same feed allowance, roughage based on Lucerne hay and wheat straw and a concentrate based on cereals and oil cakes (the control diet). The diet of two groups was fortified with cornus extract, with or without oregano and thyme essential oils, at a level 0.515 g of plant extract/essential oils per kg of concentrate. Individual milk yield was recorded weekly and feed refusals were recorded on a pen basis daily, during a six-week period of lactation. Milk samples were analyzed for the chemical composition of protein, fat, lactose and solids-not-fat constituents, somatic cell counts and total viable bacteria counts. Moreover, the milk of each group was used for yoghurt and Feta cheese production. The lipid oxidative stability, protein carbonyl content and fatty acid composition of milk, yoghurt and cheese samples were also evaluated. The results showed that the incorporation of novel plant extracts and essential oils increased the milk production per ewe. Dietary supplementation with cornus extracts and essential oils lowered lipid and protein oxidation in milk, yoghurt and cheese samples, compared to the control. However, diet supplementation with herbal extracts did not affect the fatty acid profile in milk, cheese and yoghurt or the serum biochemical parameters. In conclusion, dietary supplementation with cornus in combination with oregano and thyme has the potential to improve feed utilization and the performance of high-yield dairy Chios cross-bred ewes reared under heat stress.

## 1. Introduction

The production of sheep milk is of great importance to the agricultural sector in Mediterranean countries, and the largest part of its volume is designated for yoghurt and cheese production [1]. Several attempts have been made to manipulate rumen efficiency in order to maximize the productivity of ruminants with respect to milk production by including various feed additives in the animals’ diets, such as antibiotics, hormones, chemical growth promoters [2], enzymes and minerals [3] and plant extracts [4,5]. Feed additives such as antibiotics, hormones and other pharmaceutical substances, despite their financial benefits, have been prohibited in most countries of the world, mainly due to their potentially hazardous residues and due to the development of multi-drug resistance in bacteria [6]. The antimicrobial action of essential oils (EOs) has been known for decades and a large number of scientific studies have been carried out [7]. Recent scientific interest has focused on the addition of essential oils to ruminant nutrition in order to improve nutrient utilization efficiency and dairy product quality. Moreover, essential oils at high concentrations inhibit amino acid deamination and decrease methane production [8,9]. In view of this, the use of plant extracts and essential oils in sheep feeding should be further exploited. Additionally, milk and dairy products naturally enriched with antioxidant compounds may provide extra health benefits to the consumer [10]. Indeed, medicinal aromatic plants are used in animal nutrition and their exploitation has been greatly increased during the last decade [11].

Sheep milk is highly nutritious [1] and cheese such as feta, which is the most well-known Greek protected designation of origin product, have gained significant acceptance worldwide. It is a high-quality white-brined cheese produced from sheep milk or a mixture of sheep and goat milk (≤30%) [12]. It has a salty and slightly pungent flavor [13]. Nowadays in Greece, the majority of Feta cheese is manufactured in well-organized cheese dairies using pasteurized milk and lactic acid bacteria (LAB) as starter cultures [14]. Yoghurt is another very popular fermented dairy product which has gained significant acceptance worldwide, and its nutritional and health benefits have been well known for centuries [15]. It is an excellent source of high biological value protein, essential amino acids, calcium, phosphorus, vitamins and trace minerals such as magnesium and zinc [15].

The most commonly used plants for essential oil production in animal nutrition are *Oreganum vulgare* and *Thymus vulgaris*, two plants which are widely distributed in Greece and other Mediterranean countries. Oregano and thyme essential oils have been used in animal nutrition due to their strong antimicrobial and antiparasitic action, along with their influence on polyunsaturated fatty acid protection and the antioxidant capacity delivered to the final dairy products [16]. The use of herbal thyme as a galactagogue is known to have a beneficial effect, leading to an increase in milk production [17]. Another plant commonly found in the Mediterranean area is *Cornus* spp., with a high antioxidant activity due to its large total phenolic concentration of about 220 mg/dL gallic acid equivalents [18]. The most important phenolic compounds of cornus are anthocyanins, gallic acid and ellagic acid, which all have antioxidant and antimicrobial activity [19].

The isolation of bioactive compounds from aromatic and medicinal plants is usually performed by extraction using several solvents, most of them organic, which are harmful to the environment. Thus, it is important to explore the use of extraction methods that combine eco-friendly solvents and a high yield of bioactive compounds. Green extraction methods are using aqueous solutions, thereby avoiding organic solvents such as ethanol or methanol or energy-consuming methods such as microwave extraction or high-pressure extraction [20]. The effectiveness of aqueous solutions of cyclodextrin (CD) as an eco-friendly solvent, appropriate for extracting phenolic compounds and other phytonutrients from plant materials, such as pomegranate, olive leaves, aromatic and medicinal plants or by-products of agricultural production, has been reported [21]. The addition of CD to water augments the extraction of phenolic compounds and decrease the extraction time and temperature. The extraction of essential oils from aromatic plants can be achieved by means of steam distillation, providing products with a high phenolic content, although at a relatively high cost.

A recent major issue during the last two decades concerning dairy sheep farming in the Mediterranean is the high ambient temperatures during the summer season and the first period in autumn. High ambient temperatures, combined with high direct and indirect solar radiation, low wind speed and increased relative humidity, are commonly faced by intensively reared animals that have to survive out of their thermoneutral zone (5 °C to 25 °C) [22] and maintain lactation, while possibly suffering from heat stress [23,24]. Heat stress is a major limiting factor of dairy production in hot climates [25] that is often associated with oxidative stress, defined as the presence of a large amount of reactive oxygen species, exceeding the available antioxidant capacity of animal cells [26]. Plant extracts, rich in polyphenols, may support sheep that suffer from lipoperoxidation [27].

The objective of this study was to investigate the effect of diet supplementation with a cornus plant extract alone or in combination with oregano and thyme essential oil on the milk production and composition of dairy sheep reared under heat stress conditions, the effect on animal health, as well as the effect on the nutritional value and the characteristics of two traditional dairy products—yoghurt and feta cheese. The hypothesis of this study was that the addition of bioactive plant extracts to the sheep nutrition will lead to improved milk yield, along with increased antioxidant activity in the milk, yoghurt and cheese and the capability of animals to cope better with the adverse effects of heat stress. It must be noted that there is a lack of information on the effects of cornus, oregano and thyme in dairy sheep reared during the hot seasons.

## 2. Materials and Methods

### 2.1. Ethics Guidelines of the Animal Research

This trial was carried out according to the regulations of the Greek Public Veterinary Service and approved by the Research Committee of Aristotle University of Thessaloniki, under the project numbered 99864. The health of the animals was monitored by a veterinary surgeon; animals were routinely vaccinated against enterotoxaemia clostridial infections 2 weeks before lambing, contagious agalactia, subclinical mastitis, pasteurelosis and *Brucella melitensis*. All animals had been drenched with an antiparasitic drug containing Fenbendazole 5%, and no ewe received any antimicrobial agents during lactation. All procedures were performed by the same team members on all herds throughout the project.

### 2.2. Experimental Design and Feeding

**Figure 1:**
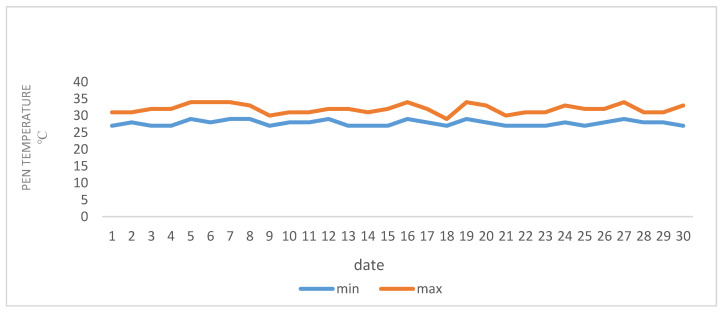
Minimum and maximum temperatures recorded on the sheep farm in July 2020.

**Figure 2:**
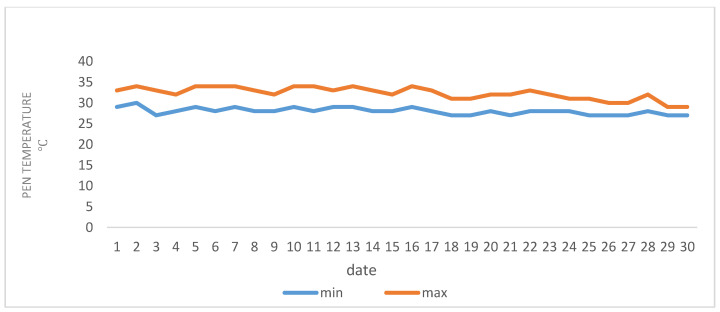
Minimum and maximum temperatures recorded on the sheep farm in August 2020.

A total of 45 lactating ewes of the Chios cross-breed were randomly selected for twin birth from a flock consisting of 150 ewes, kept on a dairy breeding sheep farm located in the area of Polykastro (Latitude: 41°00′ N; Longitude: 22°33′ E) in the northwestern part of Kilkis in Greece. The daily mean temperature during July and August (2020) ranged between 27–34 °C. Figure 1 and Figure 2 provide temperature data inside the building. The relative humidity was 65% and the average wind speed was one meter per second. The ewes were allocated into three equally sized treatment groups of 15 ewes, each consisting of 5 subgroups housed in a separate pen. Each group and subgroup was balanced for body weight (BW) three weeks after lambing, parity and body condition. The three treatment groups were fed isonitrogenous and isoenergetic diets. Group 1 (Control) was fed the basal ration with Lucerne hay and wheat straw and a concentrate containing maize, soybean meal and sunflower meal as the main constituents (Table 1). The diet of group 2 was supplemented with cornus extract at a level 0.5 g per kg of concentrate and that of group 3 with a cornus extract plus oregano and thyme essential oils, at a level of 0.5 g cornus extract, 0.01 g oregano and 0.005 g thyme essential oil per kg of concentrate, respectively (Cornus and CorOrThym groups). All plant extracts were provided by ORIZON Diatrofiki, Serres, Greece. At the start of the trial, the ewes were in the third month of their second lactation (on average two months after lamb weaning) and were acclimatized to the experimental conditions for two weeks prior to starting. The mean initial BW was 54.4 ± 1.65 kg and the trial lasted for 45 days plus acclimatization of two weeks, and the mean milk yield was 1.73 L/d. The final BW was 55.3 ± 1.44 kg. After weaning, the ewes were machine milked twice daily (at 6:30 and 17:00 h) in a 24-parallel milking parlor with individual ear-tag identification.

All ewes were fed the same total diet, which was offered twice daily in equal quantities. The daily forage allowance per ewe was 1.5 kg of Lucerne hay and 0.30 kg of wheat straw on a fresh weight basis. The concentrate feed consisted of corn grains, barley grains, wheat bran, extracted soybean meal, sunflower cake and mineral and vitamin premix (Table 1). The concentrates were fed at the level of 1.51 kg per ewe per day; thus, the total feed allowance was 3.31 kg fresh food/ewe/day. The feed allowance was kept constant throughout the experimental period, according to commercial practices. Table 1 shows the chemical analysis of the bilateral ration that was conducted according to AOAC [28]. Feed samples were collected monthly and subsequently analyzed for each group. Any orts were collected and taken into account in the calculations. Fresh water was available ad libitum.

### 2.3. Chemicals

All solvents or analytical standards such as 2,2-diphenyl-picrylhydrazyl (DPPH) stable radical, Trolox™, Folin–Ciocalteu reagent, gallic acid, nordihydroguaiaretic acid (NDGA), dimethyl sulfoxide (DMSO), trichloroacetic acid (TCA) and sodium chloride (NaCl), were purchased from Sigma-Aldrich, Chemie GmbH (Taufkirchen, Germany). For gas chromatography (GC), helium gas was purchased from Afoi Thomadaki (Thessaloniki, Greece).

### 2.4. Characterization of Cornelian Cherry Extract (Cornus mas *L.*)

#### 2.4.1. Determination of the Antiradical Activity (A_AR_)

For the antiradical activity (A_AR_) determination, a previously described protocol was used [30] with slight modifications. In brief, an aliquot of 0.025 mL of sample was added to 0.975 mL DPPH solution (100 μM in MeOH) and the absorbance was read at t = 0 and t = 30 min. Trolox™ equivalents (mM TRE) were determined based on linear regression, after plotting %ΔA_515_ against the respective known concentration of a Trolox™ solutions, where

(1) $$\%\Delta A=\frac{A_{515}^{t=0}-A_{515}^{t=30}}{A_{515}^{t=0}}\times 100$$

expressing the difference in antiradical activity, where $A_{515}^{t=30}$ is the absorbance of the sample at 515 nm after the necessary time to reach the plateau (30 min) and $A_{515}^{t=0}$ is the absorbance of the DPPH solution at 515 nm.

Results were expressed as μmol TRE/g of cornelian cherry.

#### 2.4.2. Determination of Total Polyphenol Yield (YTP)

The total phenolic content of the extracts was determined by using the Folin–Ciocalteu method [30]. The yield in total polyphenols (Y_TP_) was expressed as mg gallic acid equivalents (GAE) per 100 g of cornelian cherry extract after 180 min of incubation in dark.

#### 2.4.3. Determination of Total Monomeric Anthocyanins

The total monomeric anthocyanin content of the extract was determined according to a protocol using the pH-differential method [31,32]. Briefly, the total monomeric anthocyanin content was determined by measuring the absorbance at 510 nm and 700 nm, against distilled water. Measurements were made after dilution of the extracts in buffer solutions of pH 1.0 and pH 4.5, accordingly. The calculation was based on two equations:

(2) $$A={\left(A_{\lambda \max}-A_{700}\right)}_{pH1.0}-{\left(A_{\lambda \max}-A_{700}\right)}_{pH4.5}$$

where A_λmax_ is the absorbance of the sample at 510 nm.

(3) Total monomeric anthocyannins (mg100 g)=A∗Mw∗Df∗1000ε∗l

where M_W_ = molecular weight of cyanidin-3-glucoside (449.2 g/mol); Df = dilution factor; l = path length of the cuvette in cm; ε = molar extinction coefficient of cyanidin-3-glucoside (26,900 L/mol/cm); 1000 = conversion of g to mg. The results were expressed as mg cyanidin-3-glucoside per 100 g of cornelian cherry dry matter. All analyses were done in triplicate.

### 2.5. Essential Oil Analysis

Essential oil analyses were performed on a Shimadzu GC-2010-GCMS-QP2010 (Kyoto, Japan) system operating at 70 eV. The gas chromatograph (GC) was equipped with a split/splitless injector (230 °C) and a fused silica column INNOWAX (Santa Clara, CA, USA) (30 m × 0.25 mm, film thickness: 0.25 μm). The temperature program was from 50 °C (20 min) to 250 °C, at a rate of 3 °C/min. Helium was used as a carrier gas at a flow rate of 1.0 mL/min. The injection volume of each sample was 1 μL. The injector was set at 230 °C and operated in split mode (split ratio = 1:10), whereas the GC–MS transfer line and the ion source were set at 300 °C and 230 °C, respectively. The mass spectrometer was operated in electron ionization mode (70 eV) and full-scan mass spectra were acquired from m/z 100 to 600. The relative percentage amounts of the separated compounds were calculated from the total ion chromatogram by a computerized integrator. The identification of the components was based on comparison of their mass spectra with those of NIST21 and NIST107 [33] and by comparison with literature data [34]. Essential oils were often subjected to co-chromatography with authentic compounds.

### 2.6. Determination of Total Phenolic Content of Experimental Diets, Milk, Yoghurt and Cheese

The concentrate fed to the three different experimental groups was also analyzed for its total phenolic content according to the method of Singleton et al. (1999) [35] using the Folin–Ciocalteu assay and expressed as gallic acid equivalents (GAE). An aliquot (1 mL) of the sample or a standard solution of gallic acid (blank, 100, 200, 300, 400 and 500 µg/mL) was transferred into a 25 mL volumetric flask, containing 9 mL of distilled water. Subsequently, 1 mL of Folin–Ciocalteu phenol reagent (Merck, Darmstadt, Germany) was added to the mixture and shaken. After 5 min, 10 mL of a sodium carbonate (Na_2_CO_3_) solution (7% *w/w*) was added to the mixture. After incubating for 90 min at room temperature, the absorbance was measured against the reagent blank at 550 nm with a UV-VIS spectrophotometer (UV-1700 PharmaSpec, Shimadzu, Kyoto, Japan). For the milk, yoghurt and cheese samples, 5 g were mixed with 25 mL of an aqueous methanol solution (ratio of methanol to water: 75:15). The mixture was stirred for 30 min and centrifuged for 15 min at 7200 rpm. The upper phase was filtered through a nylon syringe filter (filter diameter: 25 mm, pore size: 0.22 μm, Frisenette, Knebel, Denmark), and analyzed for gallic acid equivalents using the procedure described above.

### 2.7. Feed and Essential Oils Interaction with DPPH

The antioxidant activity of the essential oils and the corresponding feed stuff was determined with respect to hydrogen-donating or radical-scavenging ability, according to the method of Peperidou et al. (2014) [36], using the stable radical 1.1-diphenyl-2-picrylhydrazyl (DPPH). Ten microliters of the test samples, which were dissolved in methanol (20 mg/mL stock solution), were added to a solution of DPPH (0.1 mM in DMSO). After 20 and 60 min the absorbance was recorded at 517 nm and the percentage of reducing activity (RA) was calculated and compared with the reference compound, nordihydroguaiaretic acid (NDGA).

### 2.8. Determination of Milk Yield and Composition

The milk yield of each ewe was recorded weekly by the total volume measurement method. Each sheep was recognized by their individual ear tag. Individual milk samples were analyzed for total fat, total protein, lactose, total solids, total viable counts and solid-not-fat (SNF) by means of near-infrared spectroscopy using a MilkoScan 4000 (FOSS Electric, Integrated Milk TestingTM, Hillerød, Denmark) and somatic cell counts were determined using a Fossomatic 5000 Basic (FOSS Electric, Denmark). Milk acidity was measured using a portable titrator.

### 2.9. Preparation of Yoghurt and Determination of Bacteria Culture Viability

Yoghurt was produced by sheep milk. Five liters of milk from each group (Control, Cornus and CorOrThym) were collected and heated for 8 min at 92 °C, followed by cooling to 42 °C, and the addition of the standard yogurt starter culture under aseptic conditions. Subsequently, the milk was transferred into 200-mL plastic retail containers, sealed, kept quiescent and incubated at 42 °C for 3 h. Afterwards, the samples were immediately stored at 5 °C. After 72 h, the yoghurt samples were subjected to physicochemical and organoleptic analyses. All yoghurt trials were repeated twice. Enumeration of *Lactobacilus bulgaricus* and *Streprococcus thermophilus* populations were conducted using the pour plate technique and carried out after 10 days of storage at 4 °C. An aliquot of 1 g of yoghurt was diluted in 9 mL of peptone water, containing sodium chloride (0.9% *w/v*), followed by seven sequential dilutions (10^−1^–10^−7^). A quantity of 1 mL of the diluted sample of 10^−4^ up to 10^−7^ was placed in M17 agar (Merck, Germany) and each petri was incubated at 37 °C for 72 h. For each sample, the mean numbers of colony forming units (CFU) per gram of yoghurt was estimated.

### 2.10. Feta Cheese Production and Determination of its Physico-chemical Characteristics

Fresh sheep milk was standardized to a protein-to-fat ratio of 1.0, pasteurized at 63 °C for 30 min, and then cooled to 35 °C. Cheese manufacturing was carried out according to the traditional procedure. In brief, calcium chloride solution (0.2% *w*/*v*) was added to the cheese milk. The milk was held for 30 min at 35 °C for culture maturation. Afterwards, powdered calf rennet was added to achieve coagulation in about 50 min at 35 °C. After coagulation, the curd was cut into cubes of 2 cm and left to rest for 10 min. The sliced curd was then transferred into molds, stored at 18 °C and turned every hour for 3 h for whey drainage, surface salted using dry salt and left overnight to complete whey drainage. Following, the cheeses were placed in containers with brine (7%) and kept at 18 °C for approximately 10 days for cheese ripening. Subsequently, the cheese containers were sealed and stored at 4 °C for a total ripening period of 60 days. Samples were collected on day 60 of ripening and subjected to physicochemical analyses. The pH of the cheeses was determined by a pH meter (GLP-21, CRISON Instruments SA., Barcelona, Spain). The moisture content was determined by the sea sand method, e.g., by thoroughly mixing 2 g of sample with 20 g sea sand and heating at 105 °C until it was constant in weight [37]. The sodium chloride (NaCl) content was determined according to the standard method of the International Dairy Federation [38] and expressed as salt-in-moisture concentration. Finally, total nitrogen (TN) was determined using the Kjeldahl method [28] and the fat content was determined using the Gerber van Gulik method [39].

### 2.11. Determination of the Fatty Acid Composition in Milk, Yoghurt and Feta cheese

Fatty acid methyl esters (FAMEs) were prepared according to [40] Bligh and Dyer (1959) method and [41] the International Organisation for Standardisation (ISO) (2002) as reported previously by Papaloukas et al., 2016 [42]. Analysis of FAMEs was performed on an Agilent Technologies 6890N gas chromatograph (GC), equipped with a flame ionization detector (FID) (Santa Clara, CA, USA) and a DB-23 capillary column (60 m × 0.25 mm i.d., 0.25-μm film thickness). Each peak was identified and quantified using a 37 component FAME mix (Supelco, 47885-U) and the PUFA-2, Animal source (Supelco, 47015-U) (Sigma-Aldrich, Taufkirchen, Germany). After analysis, the saturated fatty acids (SFA), monounsaturated fatty acids (MUFA), polyunsaturated fatty acids (PUFA), unsaturated fatty acids (UFA), n-3 PUFA and n-6 PUFA were further grouped together. The index for atherogenicity (AI) was determined as suggested by [43] Ulbricht and Southgate (1991), and the Δ−9 desaturase activity indexes were calculated using the following four ratios: C14:1/C14:0, C16:1/C16:0, C18:1/C18:0 and CLA/VA.

### 2.12. Determination of TBARS and Protein Carbonyls in Milk, Yoghurt and Feta Cheese

Fresh milk samples were collected on days 1, 21 and 42 of the trial. The samples were analyzed immediately. For the determination of TBARS (thiobarbituric acid reactive substances), an aliquot of 100 μL of the fresh milk sample was mixed with 500 μL of trichloroacetic acid (TCA) solution (35 % *w/v*) and 500 μL of Tris–HCl (200 mmol/L; pH 7.4), and incubated for 10 min at 20 °C. Subsequently, 1 mL of 2MNa_2_SO_4_ and 55 mM thiobarbituric acid solution were added, and the samples were incubated at 95 °C for 45 min. Finally, the absorbance was measured at 530 nm against a blank sample using an UV-VIS spectrophotometer (UV-1700 PharmaSpec, Shimadzu, Japan). Results were expressed as μmol MDA per liter of milk.

Protein carbonyl determination was based on the method of Patsoukis et al. (2004) [44]. In particular, 50 µL of a TCA solution (20% *w/v*) was added to 50 µL of sample homogenate (diluted 1:2 *v*/*v*). The mixture was then kept in an ice bath for 15 min and centrifuged at 15,000× *g* for 5 min at 4 °C. The supernatant was discarded. To the remaining pellet, 500 µL of a 2,4-dinitrophenylhydrazine (DNPH) solution (10 mmol/L in 2.5 N HCl) were added. The blank was prepared by addition of 500 µL of 2.5 N HCl to the pellet. In this assay, carbonyl formation is detected by the reaction of protein carbonyls with 2,4-dinitrophenylhydrazine (DNPH) and its subsequent conversion to 2,4-dinitrophenylhydrazone (DNP-hydrazone), which was measured at 375 nm. Calculation of protein carbonyl concentration was based on the molar extinction coefficient of DNPH (22,000 M^−1^ cm^−1^).

### 2.13. Determination of Blood Serum Parameters

The influence of the different diets on animal health indices was also evaluated by analyzing specific biochemical parameters in the blood serum. Blood samples were obtained from each ewe by jugular venipuncture into 10 mL vacuum tubes without anticoagulant with a needle, on the first and last day of the experiment. Samples were centrifuged (1600× *g* for 15 min in 4 °C), and the collected serum was frozen at −20 °C until analysis. The biochemical parameters tested in the serum were total proteins, albumins, glucose, blood urea nitrogen (BUN), creatine and gamma-glutamyl transpeptidase (γ-Gt) and they were assayed using an automated analyzer (TARGA CLIN/CHEM Analyzer, BT 1500 Biotechnica instruments Roma, Italy).

### 2.14. Statistical Analysis

Data on total milk production and composition were analyzed by ANOVA in the general linear model of the SPSS 25.00 statistical package (SPSS Inc., Chicago, IL). Individual ewes were considered replicates nested within pens; the pen was the statistical unit for the analysis. Data on total milk yield and milk, cheese, yoghurt composition, serum biochemical parameters were analyzed by means of one-way ANOVA. The homogeneity of the variances was tested using the Levene test. The Tukey multiple comparison test was carried out to assess any significant differences at a probability level of *p* < 0.05 between the experimental treatments, when a significant effect of treatment was detected by means of the ANOVA.

## 3. Results

All the animals consumed all their offered daily food allowance of 3.31 kg. Total observed feed refusals were estimated to be 0.05–1% of the total food allowance. None of the animals showed symptoms of any health affliction. The diet of the control group contained 0.18 mg GAE/g dry matter (DM). The cornus-supplemented diet contained 0.50 mg GAE/g DM; whereas the CorOrThym diet contained 0.66 mg GAE/g DM. The results of the photometric analysis of the cornelian extracts are presented in Table 2. In Table 3 the analyses of the oregano and thyme essential oils are presented. Table 4 presents the results relating to the DPPH and TP antioxidant activity of the diets and oregano and thyme essential oils.

### 3.1. Milk Yield

Milk yield followed the typical lactation curve and no interactions were observed between treatments and time (trial duration) on daily milk yield over the tested period. However, supplementation of the diet with cornus extract, with and without essential oil, seemed to improve the daily milk yield compared to the control group. The average milk yield in the first days of the trial did not differ among the groups and was found to be 1.74 L/d for the control group and 1.72 L/d for both the Cornus and CorOrThym group, respectively. On day 21 a significant increase in average milk yield of 1.76 L/d (Cornus group) and 1.92 L/d (CorOrThym group), compared to an average milk yield of 1.72 L/d for the control group, was noticed (Table 5). This pattern remained unchanged on the last day of the experiment. However, there was no connection between cornus extract and EO addition on milk composition, except for urea and somatic cell counts (SCCs), which were found to be lower in the more highly EO-supplemented groups (Table 6). Although we did not measure any changes in body composition, occasional observations did not suggest that the effects of the EO supplementation were achieved through changes in body mobilization between the treatments. In addition, BW was similar among the groups at the start and at the end of the trial.

### 3.2. Milk, Yoghurt and Feta: Composition, Fatty Acid Composition and Oxidation Status

The composition of the milk from the three investigated groups is presented in Table 6. Yoghurt and feta cheese composition was within the normal standards and no major differences were noted. The acceptance of the products was satisfactory in regard to taste and aroma.

Regarding the levels of the main fatty acids in milk, yoghurt and cheese—namely, palmitic and linoleic acids—no major differences were noted (Table 7 and Table 8).

However, it is noteworthy that the addition of Cornus and CorOrThym to the animals’ diet affected the starter culture (*Streptococcus thermophilus*) in the produced yoghurt samples; a reduction of about 2 logs of Streptococcus thermophilus was observed, compared to the control sample (Table 9). TBARS values, gallic acid equivalents and protein carbonyl levels were improved for the group fed with the cornus extract and oregano and thyme oils compared to the control group (Table 10).

### 3.3. Blood Serum Parameters

The results of the blood biochemical parameters are presented in Table 11. Diet supplementation with cornus extract with/without EO did not seem to affect the levels of total urea, creatin and gamma-glutamyl transpeptidase (γ-GT) in the serum among the experimental groups. However, the level of total protein decreased gradually in the Cornus and cornus, oregano and thyme (CorOrThym) groups towards the end of the experimental period. Interestingly, the level of total albumin was increased in the control and Cornus groups but decreased in the CorOrThym group.

## 4. Discussion

Currently, there are several commercially available EO products which are given to ruminants, including dairy animals, in many parts of the world [7,45,46,47]. In general, essential oils have a positive influence when administrated with dairy sheep rations. Adding 1.25 g/kg DM of thyme essential oil improved both ruminal fermentation and nitrogen metabolism [48]. Another study investigating the effects of oregano essential oils on sheep showed an increase in the population of three primary cellulosic bacteria and ruminal fungi, affecting the rumen fermentation process [49]. However, potential toxic effects of plant essential oils are not scarce and should be taken into consideration before feeding them to animals [50].

The plant extract used in our study included an aqueous cornus extract and a complex of cornus hydro-extract plus oregano and thyme EOs. It was found that the addition of either cornus extract alone or a mixture of cornus extract with oregano and thyme essential oils to the basal ration of dairy sheep improved milk production, milk yield and fat content, whereas differences in total protein, ash and solids-not-fat were not significant. The increasing effect of oregano and thyme on milk yield may be attributed to the galactopoietic effect of the active compounds present in the essential oils [51]. Furthermore, it is suggested that positive effects of galactagogues on milk production may be due to the decrease in circulating biogenic amines such as histamine, tryptamine and tyramine in the blood, which are known to cause the excessive release of catecholamines, leading to a reduction in milk production as well as causing indigestion by inhibiting ruminal mobility and absorption [52]. Other researchers have shown that herbal essential oils can act as galactagogues by enhancing prolactin production and releasing somatotropins, resulting in increased glucose levels in the udder and improved milk production [51,52,53,54,55].

The properties and composition of oregano and thyme have been well investigated. It was reported that their essential oils possess anti-bacterial, anti-fungal, anti-inflammatory and antihistaminic effects [54,55]. Oregano and thyme essential oils increased mammary gland development in rats, followed by a higher milk yield at different stages of lactation [56]. In addition, oregano and thyme essential oils appear to be a potential multipurpose feed additive and may be promising in improving the performance of sheep and lambs [17]. However, there are contradictory opinions in the literature regarding the effect of these plants on milk production; some researchers found that by using oregano and thyme as feed additives, milk yield was increased and that the higher levels remained rather constant [9,57]. On the other hand, it was reported that no positive effects were achieved in ruminants by supplementing their diet with oregano oil or carvacrol, oregano’s main constituent. However, it is notable that this study was conducted on dairy cows and the inclusion level of oregano oil or carvacrol was at different levels (50 mg/kg of DM intake) [58]. Additionally, the experimental period lasted for 4 weeks, and no effects were noted on nutrient utilization, ruminal fermentation characteristics, N-excretion, CH_4_-production, milk production, milk composition or milk FA profile [58]. In the literature, there are no data about the effect of cornus extracts in combination with oregano or thyme in dairy sheep. Moreover, published studies with EOs and plant extracts have been mainly focused on dairy cows [57,58]. Indeed, dietary hesperidin or naringin improved milk oxidative stability without any further side effects in milk yield, composition, coagulation properties and fatty acid profile in dairy sheep [59], whereas dietary supplementation with plant extracts rich in flavonoids resulted in increased milk yield in cows [60,61].

In contrast to oregano and thyme, few publications have described the effect of dietary cornus supplementation on ruminants. Cornelian cherry (*Cornus mas* L.) is a species of dogwood widely grown in central and south-eastern Europe and Asia. It contains phenolics, including anthocyanins, gallic acid, ellagic acid, quercetin, kaempferol and cyanin [62]. These phenolics have both antioxidant and antimicrobial properties [63,64], whereas ellagic acid also shows immune modulatory activity [64]. Quercetin, another main phenolic compound, was found to reduce inflammation and oxidation damage caused by *Helicobacter pylori* in the mucosa of guinea pigs [65]. Cornus is also rich in ascorbic acid, sugar, organic acids, flavonoids, tannins and other bioactive compounds, among which polyphenols were found in significant amounts [66]. Studies demonstrate that feeding with cornus may reduce the use of antibiotics in young animals post-weaning and the incidence of both diarrhea and mortality in rabbits [67]. Moreover, cornus in beef cattle rations displayed an improvement in DM digestibility, which primarily resulted from the enhanced digestibility of fiber (associated with increased ruminal protozoa counts by feeding cornus) and protein [18]. Another study indicated that increasing dietary cornus was beneficial for the NDF digestibility and also decreased protein degradability, providing a higher concentration of bypass protein. Moreover, in the same study, cornus contributed to the mitigation of acidosis, affecting cattle fed high-grain diets, by altering the nutrient degradability of feeds [19].

In our study, dairy sheep were reared in the hot summer months under semi-temperate climatic conditions. As the mean temperature during the trial period exceeded 28 ℃ for several hours per day, the animals may have suffered from heat stress. A study by Braun et al. [68] suggested that a blend of plant bioactive essential oils can improve feed efficiency along with calcium homeostasis in dairy cows due to the activation of specific cation-transporting proteins, resulting in an increased uptake of cations like calcium and ammonium [69]. The effects on urea and SCC could be partly an effect of milk dilution. Our findings on milk composition partly agree with a previous study [70], that showed no change in milk concentrations of fat, protein and urea N, and the milk concentrations of fat, protein and lactose content in cows fed up to 2 g/d of a supplement containing essential oil compounds such as thymol, eugenol, vanillin, guaiacol and limonene. In our study we took into account the effect of diet, season and farm management along with parity, BW and stage of lactation in order to avoid confounding the effects of specific plant extracts on milk production and milk quality, as was correctly described [71] when keeping sheep indoors and under standardized feeding conditions without any grazing. Below, we make a further attempt to elucidate the effects of cornus extract and EO supplementation.

The prohibited use of ionophores such as monensin as additives in the EU and the increased interest in ‘naturally occurring additives’ that can also be used in the ‘organic’ production of ruminant milk and meat have resulted in a great deal of interest in identifying naturally occurring compounds that positively affect ruminal fermentation and performance [59]. *Thymus vulgaris* supplementation has been found to increase milk yield and the lactation period of Sanjabi ewes [17], as well as improving the milk production of dairy goats [72]. The increase in milk yield and lactation period resulting from thyme supplementation has been associated with the remarkable vasodilator action of thyme essential oil. Particularly, its constituents like bisabolol and bisabolol oxides [72] may act as vasodilators, increasing the flow in the blood vessels which normally supply the mammary gland with all necessary components for milk production, which is helpful during heat stress periods.

Dairy animals are often exposed to stressful agents due to environmental conditions (e.g., very high or very low temperatures), management (high yield, weaning, transportation, feedlot entry) or nutrition (e.g., a high-grain diet). Stressful events have been implicated in promoting oxidative stress through the excessive production of reactive oxygen species or decreased antioxidant defenses [73]. Excessive reactive oxygen species production overwhelms antioxidant defenses, leading to the oxidative damage of biological molecules, disrupting normal metabolism and physiology. However, a reverse relationship between antioxidant intake and stress-related diseases is supported by numerous studies [74,75]. Hence, improving antioxidant capacity through feeding of cornus extract alone or in combination with oregano and thyme is expected to enhance the overall health of sheep and, thus, to improve milk performance. In support of this, a recent study, carried out using 95 control heifers and 95 heifers fed with 0.9 kg/d of cornus extract, showed that the average daily gain increased from 0.89 to 1.12 kg/d [67]. Therefore, it is hypothesized that feeding with red osier dogwood may influence the rumen microflora, immunity (intestine and body) and antioxidant status, and consequently impact feed digestion and immune response. Heat stress is one of the limiting factors affecting the production performance of Mediterranean dairy ruminants, since it evokes a series of drastic changes in animal biological functions, which include a decrease in feed intake efficiency and utilization; disturbances in the metabolism of water, protein, energy and mineral balances; enzymatic reactions; hormonal secretions and blood metabolites [74]. To avoid such situations, proper management approaches are needed. Thus, nutritional techniques can be a part of the solution; in this line, several studies have revealed that supplementing ruminants’ rations with various essential oils has shown promising results, such as a decrease in SCC in generally healthy dairy cows or goats under mild heat stress conditions [75,76,77,78].

The Chios breed of sheep is a relatively high yielding one [79] and it is mainly found in the Mediterranean basin under intensive indoor conditions that resemble those used for high-yielding dairy cows. Another crucial aim of this study was to evaluate whether a dietary mixture of cornus extract plus oregano and thyme essential oils could enhance their antioxidant properties, showing synergistic properties in simultaneous administration, as it is presented in the literature [80,81], especially during the hot summer period. As a result, diet supplementation with plant extracts and essential oils improved lipid and protein oxidative stability in milk, providing evidence that those animals could better cope with the high ambient temperatures compared to the animals on a normal diet. Moreover, yoghurt and feta cheese made from the milk of animals that were fed the plant extracts presented higher lipid and protein oxidative stability.

Based on the literature, the total anthocyanins of Cornelian cherries range between 106–850 mg cyanidin-3-glycoside/100 g dm and total phenolics between 1070–2696 mg GAE/100 g [82,83]. Cornelian cherries can be considered a good source of anthocyanins, ascorbic acid and phenolics compared to common red berries, and also represent a potential functional food ingredient [82] due to their influence on sensorial properties such as color and astringency. For this reason, analysis of the use of these cherries in foods and beverages has developed during recent decades [83] and it has been reported that total phenolic content, expressed in mg/g, may be almost doubled in a diet supplemented with cornelian extract and essential oils. It was also found that diet supplementation with a combination of cornelian extract and essential oils resulted in the highest interaction with the stable radical DPPH, followed by the cornelian extract itself, which is in agreement with the GC-MS results. Oregano and thyme EOs are rich in carvacrol and thymol, which are phenolic monoterpenoids. It seems that the activity of the essential oil is related to the presence of phenolic compounds. The main role of these components as a reducer of free radicals has been previously reported [84,85].

A great number of studies referring to the addition of essential oils to milk and milk-derived products acknowledge their beneficial effects. It is generally known that the essential oil of thyme has the potential to inhibit various types of pathogenic microorganisms. The addition of thyme and thyme essential oil leads to an increased yoghurt fermentation time, whereas the counts of lactic acid bacteria finally reach the required minimum value for dairy fermented products [86]. Another study revealed that the inclusion of thyme, marjoram and sage in yoghurt had a stimulatory effect on the starter culture and the total viable counts and that the titratable acidity was increased in Labneh cheese samples fortified with essential oil. Moreover, thyme essential oils provided a stimulatory effect on the growth of the starter culture of *Lactobacillus casei* at a dose of 0.1 mL/100 g in yoghurt preparation [86]. However, in our study we noticed that dietary incorporation of cornus extract alone or with oregano and thyme essential oils provided a significant inhibitory effect on *Streptococcus thermophilus* populations.

The observed changes in feed utilization and FA concentrations could partly be due to the antioxidative effects of the plant extracts used or to changes in rumen microbial populations. A causal explanation of how EO mixtures affect ruminal fermentation patterns through microorganism modification has not been clearly established. Evans and Martin [87] examined the effects of thymol on ruminal microorganisms. Thymol, which is a constituent of the EO mixture tested here, inhibited gram-positive bacteria (*Streptococcus bovis* at 180 μg of thymol/mL and gram-negative bacteria (*Selenomonas ruminantium*)) at 90 μg of thymol/mL. Although these in vitro concentrations are much greater than the theoretically expected concentration of the mixtures tested in our experiment, bacterial inhibition could be a potential cause of the altered values of the fatty acid profiles of milk, yoghurt and cheese observed in the current study, which may be due to differences in fermentation patterns and subsequent biohydrogenation of the unsaturated fatty acids by rumen bacteria.

## 5. Conclusions

A clear finding of the current study is that the composition of herbal mixtures can greatly influence the performance outcomes of dairy sheep reared under stress conditions. Heat stress is a major issue in Mediterranean sheep farming as it may negatively affect milk yield and composition. Although the Chios breed and its crossbreeds are maintained and adapted to temperate climatic conditions, they also suffer when environmental temperature exceeds the thermoneutral zone. The current study suggests that dietary antioxidant compounds included in cornus, oregano and thyme have a positive impact on heat stress reduction and redox homeostasis. The addition of a specific cornus mixture with EO compounds of oregano and thyme had beneficial effects on milk production, urea concentration and SCC in milk samples of dairy ewes of the Chios crossbreed in the hot summer period. The addition of cornus hydrodistillation extract, as well as oregano and thyme essential oils, improved the oxidative stability of milk, cheese and yoghurt. However, the fatty acid profile in milk, cheese and yoghurt was not affected by the dietary addition of cornus extract and oregano and thyme EOs during the third month of lactation. Future research efforts should focus on defining specific types and compositions of various plant extracts and EOs in order to optimize their level of addition, thus resulting in favorable and consistent responses and effectively utilizing their benefits.

## Figures and Tables

**Table 1:** Daily ingredient allowance and chemical composition of the diet offered to dairy ewes ^1^.

Ingredients (on Fresh Weight Basis)	Diet (g/day/ewe)
Lucerne Hay	1500
Wheat Straw	300
Corn	900
Wheat Bran	50
Sunflower Seed Meal-36	150
Soya Bean Meal-47	350
Premix ^1^ of Vitamins and Inorganic Minerals	60
Chemical Analysis (%)	-
Dry Matter	88.1
Crude Protein (N × 6.25)	17.3
Ether Extract	3.1
Crude Fiber	16.2
NDF	25.4
ADF	16.7
ADL	4.5
Ash	4.6
Starch	19.3
Sugars	6.7
Calculated Analysis	-
Calcium	16.5 g/kg
Phosphorus (total)	7.1 g/kg
PDI	102 g/kg
PDIA	58 g/kg
UF_L_	0.71 g/kg

^1^ Vitamin and mineral mix contained (per kg dry matter (DM) of concentrate): 8000 IU of vitamin A; 90 mg of vitamin E; 3000 IU of vitamin D_3_; 1.5 mg/kg biotin; 6 mg/kg niacin; 45 mg/kg choline; 0.2 mg Co; 3 mg I; 100 mg/kg Fe; 50 mg Mn; 0,45 mg Se; 150 mg Zn; 6 g of NaCl; 4 g of sulphur; 10 g of magnesium oxide; 15 g of monocalcium phosphate and 21 g of limestone. NDF = neutral detergent fiber, ADF = acid detergent fiber, ADL = acid detergent lignin, PDI = protein digestible in the small intestine, PDIA = protein digestible in the small intestine supplied by rumen–undegraded dietary protein, UF_L_ = forage unit for lactation [29].

**Table 2:** Photometric analysis of Cornelian cherry extract.

Composition	Extract
Total phenolics (mg/100 g) *	7611 ± 623
Antiradical activity (μmol TRE/g) **	58,057 ± 741
Total monomeric anthocyanins (mg/100 g) ***	382 ± 15

* as gallic acid. ** TRE = Trolox equivalent. *** cyanidin-3-glucosid.

**Table 3:** Composition (%) of oregano and thyme essential oil.

Compounds ^a^	%	Compounds ^a^	%
Oregano	-	Thyme	-
α-Pinene	0.77	α-Pinene	1.84
α-Thujene	0.52	α-Thujene	0.94
Camphene	0.07	Camphene	1.03
β-Pinene	0.09	β-Pinene	0.36
α-Phellandrene	0.14	δ 3-Carene	0.11
β-Myrcene	1.26	α-Phellandrene	0.21
α-Terpinene	1.02	β-Myrcene	1.87
D-Limonene	0.17	α-Terpinene	2.07
γ-Terpinene	7.57	D-Limonene	0.68
3-Octanone	0.27	Eucalyptol	1.64
p-Cymene	7.29	γ-Terpinene	12.55
3-Octanol	0.08	p-Cymene	38.34
1-Octen-3-ol	1.01	Terpinolene	0.18
*cis*-Sabinenehydrate	0.68	3-Octanol	0.20
*trans*-Sabinenehydrate	0.34	1-Octen-3-ol	1.19
Linalool	0.48	*cis*-Sabinene Hydrate	1.05
4-Octanol	0.05	Camphor	0.43
Bornyl Acetate	0.06	*trans*-Sabinene Hydrate	0.30
β-Caryophyllene	2.24	Linalool	3.63
Thymol Methyl Ether	0.06	Bornyl Acetate	0.17
Dihydrocarvone	0.09	β-Caryophyllene	1.64
1-Terpinen-4-ol	0.94	Thymol Methyl Ether	0.85
Carvacrol Methyl Ether	0.50	1-Terpinen-4-ol	0.48
α-Caryophyllene	0.18	Carvacrol Methyl Ether	0.63
Borneol	0.95	Borneol	1.72
β-Bisabolene	0.87	δ-Cadinene	0.21
Germacrene D	0.15	Caryophyllene Oxide	0.13
Caryophyllene oxide	0.14	Thymol	20.32
Thymol	13.51	Hinesol	0.14
Hinesol	0.33	Carvacrol	2.52
Carvacrol	57.23	Apiol	0.16
Apiol	0.41	Ledol	0.04

^a^ Compounds are listed in order of elution from an INNOWAX capillary column.

**Table 4:** Photometric analysis of oregano and thyme essential oils and sheep diets.

Sample ^2^	DPPH (20 mg/mL)		TP *
	20 min	60 min	GA
Oregano essential oil (EO)	87.70	93.55	837.93
Thyme EO	24.15	27.87	535.0
Feed Control	46.23	44.56	18.02
Feed Cornus	75.27	85.87	50.25
Feed CorOrThym	62.36	69.56	66.32
NDGA ^1^	81	93	

^1^ Results are given as means of groups (*n* = 5 = subgroups); ^2^ Control, Cornus and CorOrThym represent diets supplemented with corresponding plant extracts; * TP = total phenolics as gallic acid (GA) equivalents (mg/L); ^1^ NDGA = nordihydroguaiaretic acid.

**Table 5:** Effects of diet supplementation with cornus extract and oregano and thyme essential oils on milk yield.

Milk Yield ^1^	Control ^2^	Cornus	CorOrThym	SEM ^3^	*p*-Value
Day 0	1.74	1.72	1.72	0.03	NS
Day 21	1.72 ᵇ	1.76 ᵃᵇ	1.92 ᵃ	0.04	0.04
Day 42	1.71 ᵇ	1.78 ^ab^	1.92 ᵃ	0.03	0.04

^1^ Results are given as means of groups (*n* = 5 = subgroups); ^2^ Control, Cornus and CorOrThym represent groups of ewes fed a basal diet supplemented with corresponding plant extracts; ^a,b^ values with a superscript in common in the same line do not differ significantly. ^3^ SEM = standard error of the mean. NS = not significant.

**Table 6:** Effects of diet supplementation with cornus extract and oregano and thyme essential oils on milk composition and somatic cell counts (SCCs) of ewes during lactation.

Composition ^1^	Control ^2^	Cornus	CorOrThym	SEM ^4^	*p*-Value
Day 0					
Protein (%)	5.65	5.61	5.62	0.26	NS
Fat (%)	5.85	5.88	5.84	0.35	NS
Lactose (%)	4.77	4.77	4.78	0.22	NS
Ash (%)	0.85	0.89	0.86	0.56	NS
SNF ^3^ (%)	11.24	11.21	11.23	0.38	NS
SCC (×10^3^/mL)	26	24.0	27	25.6	NS
pH	6.72	6.71	6.71	0.05	NS
Acidity	22.1	22.2	22.5	0.36	NS
Urea (mg/100 mL)	58.4	52.1	59.3	2.95	NS
Total viable counts (×10^3^/mL)	160	160	160	15.3	NS
Day 42					
Protein (%)	5.85	5.89	5.95	0.25	NS
Fat (%)	5.95 ^b^	6.16 ^b^	6.39 ^a^	0.39	0.04
Lactose (%)	4.82	4.82	4.88	0.26	NS
Ash (%)	0.85	0.89	0.86	0.55	NS
SNF ^3^ (%)	11.31	11.32	11.31	0.61	NS
SCC (×10^3^/mL)	36 ^a^	33 ^b^	32 ^b^	22.4	0.01
pH	6.72	6.71	6.71	0.04	NS
Acidity	22.1	22.2	22.5	0.32	NS
Urea (mg/100 mL)	48.3 ^a^	32.1 ^b^	29.4 ^b^	2.08	0.004
Total viable counts (×10^3^/mL)	200 ^a^	140 ᵇ	130 ᵇ	13.5	0.005

^1^ Results are given as means of groups (*n* = 5 = subgroups); ^2^ Control, Cornus and CorOrThym represent groups of ewes fed basal diet supplemented with corresponding plant extracts; ^3^ SNF = solids-not-fat content of milk. SCC =somatic cell counts, ^a,b^ values with a superscript in common in same line do not differ significantly. ^4^ SEM = standard error of the mean. NS = not significant.

**Table 7:** Effects of diet supplementation with cornus extract and oregano and thyme essential oils on fatty acid profile of milk (% of total identified fatty acids).

*Fatty Acids* ^1^	Control ^2^	Cornus	CorOrThym	SEM ^4^	*p*-Value
*C4:0*	1.01	1.00	0.91	0.03	NS
*C6:0*	1.34	1.33	1.37	0.07	NS
*C8:0*	2.11	2.03	2.13	0.14	NS
*C10:0*	9.60	9.36	9.48	0.65	NS
*C11:0*	0.37	0.37	0.39	0.03	NS
*C12:0*	7.08	7.43	6.96	0.50	NS
*C13:0*	0.11	0.10	0.10	0.01	NS
*C14:0*	15.84	18.01	15.96	1.25	NS
*C14:1*	0.52	0.74	0.60	0.06	NS
*C15:0*	0.98	0.85	0.91	0.05	NS
*C15:1*	0.22	0.16	0.17	0.01	NS
*C16:0*	29.44	31.02	29.07	1.74	NS
*C16:1*	0.85	1.21	1.17	0.10	NS
*C17:0*	0.52	0.39	0.50	0.04	NS
*C17:1*	0.22	0.13	0.22	0.01	NS
*C18:0*	8.69	6.08	7.08	1.17	NS
*18:1 trans-11*	0.33	0.19	0.25	0.07	NS
*C18:1 n-7 cis-VA*	0.59	0.48	0.59	0.31	NS
*18:1cis cis-9*	15.87	15.23	17.16	1.75	NS
*18:2 n-6 trans*	0.62	0.69	0.88	0.34	NS
*18:2 n-6 cis*	2.27	1.91	2.22	0.18	NS
*18:3n6*	0.11	0.12	0.16	0.02	NS
*18:3n3*	0.80	0.57	0.88	0.16	NS
*CLAcis-9, trans-11*	0.35	0.48	0.52	0.12	NS
*C20:0*	0.03	0.00	0.12	0.01	NS
*C21*	0.02	0.05	0.07	0.02	NS
*C20:2*	0.11	0.08	0.12	0.02	NS
*SCFA (C4-C11 sat)*	14.43	14.09	14.29	0.86	NS
*MCFA (C12-C16 sat)*	53.45	57.41	53.00	3.40	NS
*LCFA (C17-C21 sat)*	9.26	6.52	7.76	1.26	NS
*PUFA*	4.26	3.84	4.78	0.81	NS
*MUFA*	18.60	18.14	20.17	2.05	NS
*UFA*	22.86	21.99	24.95	2.80	NS
*SFA*	77.14	78.02	75.05	2.79	NS
Al((C12:0 + 4 * C14:0 + C16:0)/UFA)	4.37	5.03	4.00	0.48	NS
*C14:1/C14:0 ^3^*	0.03	0.04	0.04	0.01	NS
*C16:1/C16:0*	0.03	0.04	0.04	0.01	NS
*C18:1/C18:0*	1.86	2.54	2.46	0.01	NS
*C18:2cis9,trans-11/C18:1 trans11*	0.60	0.99	0.88	0.07	NS
*SFA/UFA*	3.38	3.55	3.01	0.30	NS

SCFA: short chain fatty acids; MCFA: medium chain fatty acids; LCFA: long chain fatty acids; PUFA: polyunsaturated fatty acids; MUFA: monounsaturated fatty acids; SFA: saturated fatty acids; UFA: unsaturated fatty acids; ^1^ results are given as means of groups (*n* = 5 = subgroups); ^2^ Control, Cornus and CorOrThym represent groups of ewes fed a basal diet supplemented with corresponding plant extracts; ^3^ the Δ−9 desaturase activity indexes were calculated using the following four ratios: C14:1/C14:0, C16:1/C16:0, C18:1/C18:0 and C18:2cis9,trans-11/C18:1 trans11; ^4^ SEM = standard error of the mean. NS = not significant.

**Table 8:** Effects of diet supplementation with cornus extract and oregano and thyme essential oils on fatty acid profile of yoghurt and Feta cheese (% of total identified fatty acids).

Fatty Acids ^1^	Control ^2^	Cornus	CorOrThym	Control	Cornus	CorOrThym	SEM ^4^	*p*-Value
-	Yoghurt			Feta Cheese			*-*	-
C4:0	0.86	1.14	1.37	0.77	0.85	0.76	0.23	NS
C6:0	1.04	1.27	1.32	1.30	1.43	1.27	0.07	NS
C8:0	1.67	1.97	2.03	1.78	2.32	2.06	0.09	NS
C10:0	7.12	8.52	8.31	7.76	9.81	8.70	0.39	NS
C11:0	0.29	0.31	0.34	0.32	0.39	0.22	0.01	NS
C12:0	5.72	6.49	6.02	5.70	7.17	6.72	0.25	NS
C13:0	0.14	0.13	0.14	0.12	0.15	0.13	0.00	NS
C14:0	14.70	15.42	14.30	15.27	15.69	14.76	0.35	NS
C14:1	0.45	0.32	0.39	0.44	0.32	0.38	0.03	NS
C15:0	1.40	1.03	1.16	1.19	0.95	1.05	0.04	NS
C15:1	0.26	0.16	0.18	0.27	0.18	0.19	0.01	NS
C16:0	30.92	28.98	27.52	32.02	27.55	28.12	0.84	NS
C16:1	1.41	0.63	1.33	1.42	0.98	1.06	0.13	NS
C17:0	0.65	0.50	0.44	0.66	0.52	0.58	0.02	NS
C17:1	0.29	0.17	0.22	0.26	0.20	0.24	0.01	NS
C18:0	7.83	8.33	7.87	7.28	7.92	8.36	0.59	NS
18:1 trans-11	0.33	0.32	0.54	0.36	0.36	0.51	0.04	NS
C18:1 n-7 cis-VA	0.77	0.64	1.37	0.94	0.74	1.16	0.13	NS
18:1 *cis-9*	18.90	18.77	18.50	16.79	17.43	17.69	0.66	NS
18:2 n-6 trans	0.75	0.62	1.16	0.62	0.66	0.85	0.17	NS
18:2 n-6 cis	2.45	2.53	3.14	2.36	2.70	2.71	0.16	NS
18:3n6	0.29	0.10	0.16	0.20	0.22	0.28	0.03	NS
18:3n3	0.82	0.76	0.95	1.02	0.71	0.85	0.19	NS
CLAcis-9, trans-11	0.66	0.67	0.82	0.80	0.53	0.86	0.05	NS
C20:0	0.08	0.11	0.18	0.09	0.08	0.18	0.10	NS
C21	0.09	0.08	0.11	0.09	0.05	0.17	0.02	NS
C20:2	0.13	0.01	0.15	0.20	0.11	0.16	0.03	NS
SCFA	10.98	13.21	13.36	11.92	14.79	13.00	0.62	NS
MCFA	52.88	52.04	49.15	54.29	51.51	50.78	1.34	NS
LCFA	8.63	9.03	8.59	8.11	8.57	9.29	0.61	NS
PUFA	5.09	4.70	6.38	5.20	4.93	5.71	0.45	NS
MUFA	22.41	21.02	22.53	20.48	20.21	21.22	0.74	NS
UFA	27.50	25.72	28.91	25.68	25.14	26.93	1.13	NS
SFA	72.50	74.28	71.10	74.32	74.86	73.07	1.13	NS
Al(C12:0 + 4 * C14:0 + C16:0)/UFA)	3.47	3.78	3.14	3.85	3.88	3.49	0.20	NS
*C14:1/C14:0* ^3^	0.03	0.02	0.03	0.03	0.02	0.03	0.01	NS
*C16:1/C16:0*	0.05	0.02	0.05	0.04	0.04	0.04	0.01	NS
*C18:1/C18:0*	2.46	2.29	2.42	2.36	2.25	2.18	0.12	NS
*C18:2cis9,trans-11/C18:1 trans11*	0.85	1.05	0.60	0.85	0.71	0.74	0.01	NS
SFA/UFA	2.64	2.89	2.46	2.89	2.98	2.71	0.01	NS

SCFA: short chain fatty acids; MCFA: medium chain fatty acids; LCFA: long chain fatty acids; PUFA: polyunsaturated fatty acids; MUFA: monounsaturated fatty acids; SFA: saturated fatty acids; UFA: unsaturated fatty acids; ^1^ results are given as means of groups (*n* = 5 = subgroups); ^2^ Control, Cornus and CorOrThym represent groups of ewes fed a basal diet supplemented with corresponding plant extracts; ^3^ the Δ−9 desaturase activity indexes were calculated using the following four ratios: C14:1/C14:0, C16:1/C16:0, C18:1/C18:0 and C18:2cis9,trans-11/C18:1 trans11; ^4^ SEM = standard error of mean. NS = not significant.

**Table 9:** Effects of diet supplementation with cornus extract and oregano and thyme essential oils on yoghurt bacteria populations.

Yoghurt ^1^	Control ^2^	Cornus	CorOrThym	SEM ^3^	*p*-Value
*Streptococcus thermophilus*	1.3 × 10^9 a^	2.6 × 10^7 b^	3.9 × 10^7 b^	0.10	0.011
*Lactobacillus bulgaricus*	ND ^4^	ND	ND	-	-

^1^ Results are given as means of groups (*n* = 5 = subgroups); ^2^ Control, Cornus and CorOrThym represent groups of ewes fed a basal diet supplemented with corresponding plant extracts; ^a,b^ values with a superscript in common in same line do not differ significantly; ^3^ SEM = standard error of the mean; ^4^ ND = not detected.

**Table 10:** Effects of diet supplementation with cornus extract and oregano and thyme essential oils on antioxidant status of milk, yoghurt and Feta cheese.

Parameter ^1^	Control ^2^	Cornus	CorOrThym	SEM ^3^	*p*-Value
Milk					
TBARS (ng/mL)	0.14	0.14	0.13	0.17	NS
Protein carbonyls (ng/mL)	23.63 ^a^	20.90 ^a^	11.81 ^b^	0.68	0.02
TP *	0.05 ^b^	0.05 ^a^	0.58 ^a^	0.06	0.01
Yoghurt					
TBARS (ng/mL)	0.18	0.14	0.14	0.48	0.08
Protein carbonyls (ng/mL)	11.81 ^a^	9.54 ^a^	4.09 ^b^	0.68	0.01
TP *	0.03 ^b^	0.08 ^a^	0.08 ^a^	0.05	0.01
Feta Cheese					
TBARS (ng/mL)	2.30	0.53	0.48	0.48	0.02
Protein carbonyls (ng/mL)	30.90 ^a^	10.90 ^b^	7.72 ^b^	0.68	0.005
TP *	0.02 ^c^	0.09 ^b^	0.13 ^a^	0.03	0.003

^1^ Results are given as means of groups (*n* = 5 = subgroups); ^2^ Control, Cornus and CorOrThym represent groups of ewes fed a basal diet supplemented with corresponding plant extracts; * as gallic acid equivalents (ng/mL); ^a,b,c^ values with a superscript in common in the same line do not differ significantly; ^3^ SEM = standard error of the mean. NS = not significant.

**Table 11:** Effects of diet supplementation with cornus extract and oregano and thyme essential oils on blood parameters ^1^ of dairy ewes on day 1 and day 42.

Serum ^1^	Control ^2^	Cornus	CorOrThym	SEM ^10^	*p*-Value
Day 1					
GLU ^3^ (g/dL)	14.8	12.8	18.8	1.57	NS
TP ^4^ (g/dL)	10.28	11.22	10.94	0.24	NS
ALB ^5^ (g/dL)	4.32 ^b^	4.62 ^b^	5.06 ^a^	0.11	0.02
UR ^6^ (g/dL)	66.4	53.4	66.6	3.42	NS
CR ^7^ (g/dL)	0.83	0.84	0.76	0.02	NS
γ-GT ^8^ (g/dL)	53	57.1	37.06	4.39	NS
TBIL ^9^ (g/dL)	0.79	1.11	1.45	0.21	NS
Day 42					
GLU ^3^ (g/dL)	53.2	40.36	28.4	4.91	NS
TP ^4^ (g/dL)	11.76	10.44	7.32	0.85	0.08
ALB ^5^ (g/dL)	5.24	5.9	3.66	0.52	NS
UR ^6^ (g/dL)	67.6	78.4	65.4	6.74	NS
CR ^7^ (g/dL)	1.18 ^ab^	1.42 ^a^	0.89 ^b^	0.08	0.01
γ-GT ^8^ (g/dL)	32.13	22	45.2	8.03	0.07
TBIL ^9^ (g/dL)	3.79	7.23	1.36	1.06	0.06

^1^ Results are given as means of groups (*n* = 5 = subgroups); ^2^ Control, Cornus and CorOrThym represent groups of ewes fed a basal diet supplemented with corresponding plant extracts; ^a,b^ values with a superscript in common in the same line do not differ significantly. ^3^ GLU = glucose content of blood serum; ^4^ TP = total proteins of blood serum; ^5^ ALB = albumin content of blood serum; ^6^ UR = urea of blood serum; ^7^ CR = creatinine of blood serum; ^8^ γ-GT = gamma-glutamyltransferase of blood serum; ^9^ TBIL = total bilirubin of blood serum; ^10^ SEM = standard error of the mean. NS = not significant.

## Data Availability

The data presented in this study are available on request from the corresponding author. The data are not publicly available due to privacy of Orizon Diatrofiki.
